# Catechins as Tools to Understand the Molecular Basis of Neurodegeneration

**DOI:** 10.3390/molecules25163571

**Published:** 2020-08-06

**Authors:** Karla Martinez Pomier, Rashik Ahmed, Giuseppe Melacini

**Affiliations:** 1 Department of Chemistry and Chemical Biology, McMaster University, Hamilton, ON L8S 4M1, Canada; martik39@mcmaster.ca; 2 Department of Biochemistry and Biomedical Sciences, McMaster University, Hamilton, ON L8S 4M1, Canada; ahmedrt@mcmaster.ca

**Keywords:** Alzheimer’s Disease, amyloid beta, catechin, EGCG, NMR, α-synuclein

## Abstract

Protein misfolding as well as the subsequent self-association and deposition of amyloid aggregates is implicated in the progression of several neurodegenerative disorders including Alzheimer’s and Parkinson’s diseases. Modulators of amyloidogenic aggregation serve as essential tools to dissect the underlying molecular mechanisms and may offer insight on potential therapeutic solutions. These modulators include green tea catechins, which are potent inhibitors of amyloid aggregation. Although catechins often exhibit poor pharmacokinetic properties and bioavailability, they are still essential tools for identifying the drivers of amyloid aggregation and for developing other aggregation modulators through structural mimicry. As an illustration of such strategies, here we review how catechins have been used to map the toxic surfaces of oligomeric amyloid-like species and develop catechin-based phenolic compounds with enhanced anti-amyloid activity.

## 1. Introduction

Neurodegenerative diseases, including Alzheimer’s disease (AD) and Parkinson’s disease (PD), have been associated with the accumulation of amyloid-like aggregates formed by intrinsically disordered proteins (IDPs) [1]. IDPs, such as the amyloid beta-peptide (Aβ) and α-synuclein (αSyn) linked to AD and PD, respectively, lack a stable three-dimensional structure under physiological conditions, and if not tightly regulated they often self-assemble into cytotoxic aggregates [2]. The mechanism underlying the aggregation of IDPs is not fully understood, but several factors such as pH, oxidation, metal ions, and ligand binding are known to significantly affect IDP self-association [3,4,5,6,7].

Ligands that perturb the self-association of IDPs offer a potential therapeutic strategy for the treatment of amyloidogenic neurodegenerative diseases. Inhibitors of amyloid aggregation range from small molecules, peptides, and antibodies to plasma proteins such as human serum albumin and transferrin [8,9,10,11,12,13,14,15,16,17,18,19,20,21,22]. Among the low MW ligands, polyphenolic compounds naturally found in green tea, also termed catechins, have attracted considerable interest for their ability to remodel neurotoxic oligomers into non-neurotoxic assemblies [23,24,25,26,27]. Catechins have also shown inhibitory effects on Aβ aggregation in model organisms such as *Caenorhabditis elegans* [28,29]. In addition, catechins exhibit other neuroprotective effects, including antioxidant and anti-inflammatory properties [30,31,32].

Although the neuroprotective, antioxidant, and anti-inflammatory properties make catechins a potential therapeutic lead for the treatment of neurodegenerative diseases, their typically poor pharmacokinetics, including variable bioavailability and instability, limit their effectiveness as drug leads [33]. Nevertheless, catechins remain useful biochemical tools for probing the mechanisms underlying the formation of toxic aggregates [34]. Here, we focus on summarizing the use of catechins as tools for mapping the determinants of Aβ and αSyn amyloid toxicity through examples from the literature and our own work [21,27,34,35,36]. In addition, we also present other studies where the previous knowledge of catechin-based amyloid inhibition was useful for the synthesis of new phenolic compounds [12,37,38]. This manuscript does not claim to be exhaustive and for an account of other mechanistic studies on catechins and amyloids, the reader is referred to other excellent reviews in the field [32,39,40,41,42,43,44].

## 2. Catechins: Neuroprotective Effects and Therapeutic Challenges

### 2.1. Neuroprotective Effects

**Figure 1:**
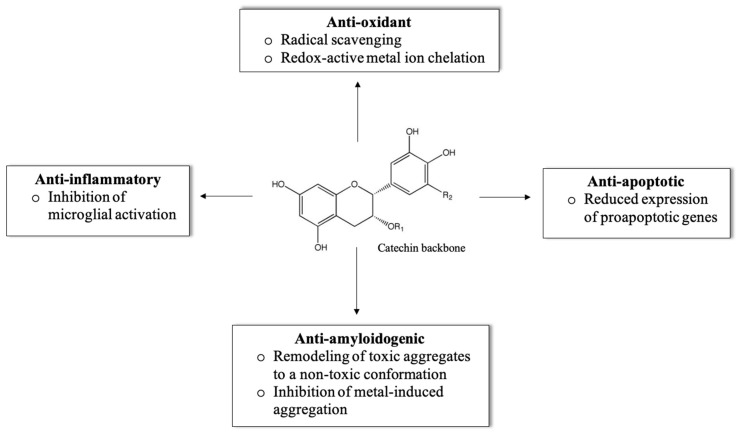
Neuroprotective effects of catechins. Catechins exert neuroprotective effects through several mechanisms including antioxidant, anti-inflammatory, anti-apoptotic, and anti-amyloidogenic responses.

The role of green tea catechins in neuroprotection have been widely studied [44,45,46,47,48,49,50,51,52]. Green tea contains several bioactive components including catechins, which contribute around 30% of the dry weight of green tea leaves [45]. The four most abundant catechins present in green tea are (–)-epicatechin (EC), (–)-epigallocatechin (EGC), (–)-epicatechin gallate (ECG), and (–)-epigallocatechin gallate (EGCG) [46,47,48,49,50,51,52,53]. Since EGCG constitutes about 65% of all catechins in green tea, it is thought to be responsible for most of its biological effects, including neuroprotection [54]. EGCG is well known not only for its anti-oxidative properties through radical scavenging and metal ion chelation [55,56,57,58,59,60], but also for its anti-inflammatory properties through inhibition of microglial activation and anti-amyloidogenic potency through oligomer remodeling (Figure 1) [21,23,24,25], [39,61,62,63,64,65,66,67]. For this review, we will focus on the neuroprotective effects related to the remodeling of Aβ and αSyn aggregates.

### 2.2. Challenges in the Therapeutic Use of Catechins

Although ample evidence supports the neuroprotective effects of green tea catechins, most in vitro studies show that the ameliorative effects of catechins require effective concentrations in the sub-mM range (1–100 µM) [68]. However, the plasma concentration of catechins typically reaches peak values only in sub-to-low µM range after oral administration [69]. One of the reasons behind the poor bioavailability of catechins is their instability in neutral and alkaline environments, conditions under which catechin auto-oxidation is enhanced [70,71,72,73,74,75,76]. Moreover, after passing through salivary and gastric fluids, they show low digestive recovery [77,78,79,80]. Catechins also have limited membrane permeability across the intestine and can be enzymatically processed or degraded by microorganisms [40,68,78,81]. Even if the disadvantages of oral administration can be overcome by administering catechins intravenously, catechins are still partially subject to degradation [69]. Another major challenge in the therapeutic application of catechins is that polyphenols are sensitive to thermal processing, light, and oxidants [82,83]. In addition, polyphenols may lack specificity. The hydroxyl groups of polyphenols may dissociate into negatively charged phenolates, which are known to interact with positive charged amino acids, such as lysine and arginine, commonly found on protein surfaces [84].

Despite the challenges in the therapeutic use of catechins, their interaction with amyloidogenic IDPs, such as the Aβ peptide, and their toxic aggregates should not be dismissed. Studying the interaction of the most abundant green tea catechin, EGCG, and its analogs with Aβ toxic oligomers, is a valuable means to elucidate the molecular mechanisms underlying the toxicity of Aβ oligomers and dissect the determinants of oligomer toxicity. For example, using a library of catechins it was possible to map toxic surfaces within soluble Aβ assemblies and such surfaces are anticipated to enable the design of new anti-amyloid therapeutics [21,24,34,38].

## 3. Mechanisms of Amyloid Inhibition by Catechins

The interactions between EGCG and amyloidogenic proteins, such as Aβ, αSyn, islet amyloid polypeptide (IAPP), huntingtin, tau, and immunoglobulin light chains [25,27,36,85,86,87,88], reveal a common mechanism to account for EGCG’s inhibitory effect on aggregation. According to this model, EGCG not only redirects the self-association of amyloidogenic peptides into off-pathway, non-toxic aggregates (Figure 3b) [21,24,25,36,39], but also remodels pre-formed amyloid fibrils into smaller, non-toxic aggregates without their disassembly into monomers [24,89,90,91]. Notably, the EGCG-induced aggregates exhibit reduced seeding capacity, i.e., the ability of amyloidogenic aggregates to incorporate monomeric subunits and build into amyloid structures [36,85,86,87,88,89,92].

**Figure 2:**
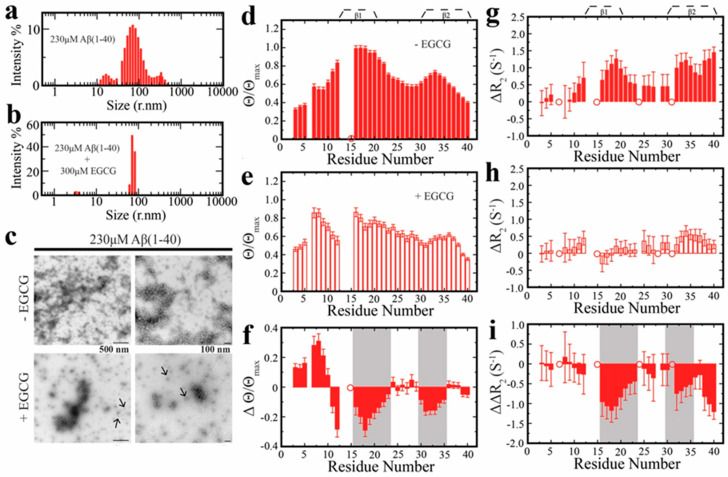
Binding of EGCG affects the recognition of Aβ_40_ monomers by Aβ_40_ protofibrils. (**a** and **b**) Size distribution of Aβ monomers and protofibrils confirmed by dynamic light scattering (DLS) in the absence (**a**) and presence of EGCG (**b**). (**c**) Negative stain electron microscopy images of samples (**a**) and (**b**). The sample containing EGCG presents oligomers with a quasi-spherical morphology as indicated by black arrows. (**d**) Dark exchange saturation transfer (DEST) differences (Θ) of each residue measured for the sample used in (**a**). The open circles indicate residues affected by overlap of the peaks. (**e**) Same as (**d**) but for the sample containing EGCG used in (**b**). (**f**) Differences between the Θ/Θ_max_ ratios for the samples in (**d**) and (**e**). (**g**) Differential ^15^N transverse relaxation rates, ∆R_2_, between the sample used in (**a**) and a monomeric reference sample. (**h**) Same as (**g**) but instead the reported ∆R_2_ is between the sample used in (**b**) and the monomeric reference sample. (**i**) Difference between the ∆R_2_ values reported in (**g**) and (**h**). In panels (**f**) and (**i**) the areas highlighted in gray represent regions with negative ∆Θ/Θ_max_ and ∆∆R_2,_ respectively, that partially overlap with the *β*-strands found is cross-*β* structures [21] Reprinted with permission from The American Chemical Society. **2017**, 139, 39, 13720–13734. Copyright (2017) American Chemical Society.

High-Resolution mechanistic insight into the EGCG remodeling of Aβ oligomers was provided by both solution and solid-state NMR [21,92]. EGCG binds to Aβ oligomers at multiple equivalent and independent sites with a per-site affinity in the sub-200 μM range. Consequently, EGCG can interfere with the on-off exchange of monomeric Aβ with the protofibril surface. Dark-state exchange saturation transfer (^15^N-DEST) spectroscopy and comparative analysis of the ^15^N R_2_ relaxation rates uncovered that monomer-protofibril contacts at the β_1_ strand (residues 15–22), which are critical for self-association, are weakened in the presence of EGCG (Figure 2) [21]. However, at the same time EGCG enhances monomer-protofibril interactions at the N-terminal region (Figure 2). These dichotomous EGCG-induced effects explain how EGCG remodels mature amyloid assemblies into smaller, non-toxic aggregates without the release of transient Aβ monomers. In addition, the authors showed that upon binding to EGCG, Aβ_40_ oligomers become less solvent-exposed. This EGCG-induced shielding effect is likely due to the occupancy of the oligomer surface by EGCG bound at different sites. Shielding of the oligomer surface is concomitant with the reduction in exposure of hydrophobic residues, which is a key determinant of oligomer cytotoxicity [93]. Overall, these findings provide a general mechanism for the neuroprotective effects of EGCG arising from the remodeling of toxic oligomers into non-toxic species.

**Figure 3:**
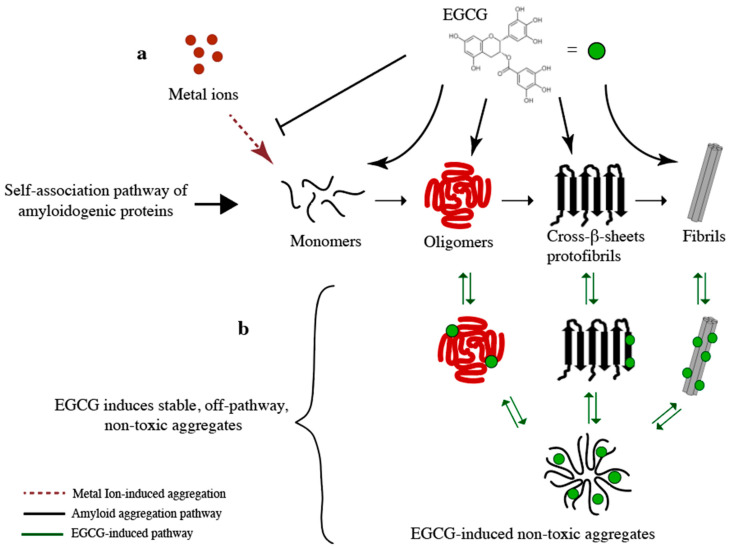
Common mechanisms of amyloid inhibition by EGCG. (**a**) Metal-induced amyloid aggregation is inhibited by the chelating properties of EGCG; (**b**) EGCG binds to different species of amyloidogenic proteins and generates off-pathway non-toxic aggregates. The stoichiometry of EGCG-protein complex is not fully known. The stoichiometry of the complexes shown in this figure is included for illustrative purposes only.

In addition to the direct binding and remodeling of oligomeric species, another mechanism by which catechins inhibit amyloid aggregation is through chelation of metal ions, which are known enhancers of amyloid fibrillization (Figure 3a) [94]. Metal ions such as Cu (II), Zn (II), and Fe (II) enhance fibrillization of amyloid proteins, such as Aβ and ⍺Syn [95,96,97,98]. Natural phenolic compounds such as EGCG are known to bind these metal ions and inhibit metal-enhanced amyloid aggregation [23], [94]. For example, a study by Teng et al. found that EGCG was able to interfere with the Cu (II)-induced fibrillation of ⍺Syn and reduce cell toxicity [94]. Further building on EGCG as a metal chelator, Hyung et al. examined the interactions of the phenolic compound with metal-free and metal-associated Aβ species [23]. They found that EGCG disrupted more effectively metal-mediated than metal-independent Aβ aggregation pathways. This observation led to the notion that EGCG generates off-pathway Aβ intermediates preferentially in the presence of Cu (II) and Zn (II) ions. The same group also showed that bifunctional small molecules with metal chelating and Aβ binding moieties are effective in reducing metal-induced Aβ aggregation and neurotoxicity [99,100]. In general, these studies elucidate how the most abundant catechin, EGCG, reduces the cytotoxicity of Aβ oligomers and the resulting mechanisms provide a foundation to develop EGCG-based amyloid inhibitors. As an illustrative example, in the following sections we describe the characterization of the structural determinants of catechin binding to amyloid oligomers and the development of new synthetic polyphenol-based amyloid inhibitors [37,38].

## 4. Structural Determinants of Catechin Binding to Amyloid Oligomers

The previous section describes the mechanisms of amyloid inhibition by catechins, emphasizing the remodeling of toxic peptide oligomers. However, the catechin structural features critical for such remodeling activity are yet to be fully discussed. Sironi et al. combined NMR spectroscopy, transmission electron microscopy and circular dichroism to dissect the specific catechin structural elements responsible for the interaction with amyloidogenic peptides [12]. To this end, Sironi et al. extracted the major components of green tea, and performed an initial screen using NMR spectroscopy to identify ligands for Aβ_42_, PrP_106–126_, and ataxin-3 (AT3Q55) oligomers. Using saturation transfer difference (STD) NMR the authors identified select components of the green tea extract that interact with the peptide oligomers. The STD data identified EGCG and possibly EGC as binders of Aβ_42_, PrP_106–126_, and AT3Q55. Although previous studies reported the binding of both catechins to Aβ_42_ and PrP_106–126_, the binding to AT3Q55 was not reported previously.

### 4.1. Mapping the EGCG Epitopes for Binding to Amyloid Proteins

**Figure 4:**
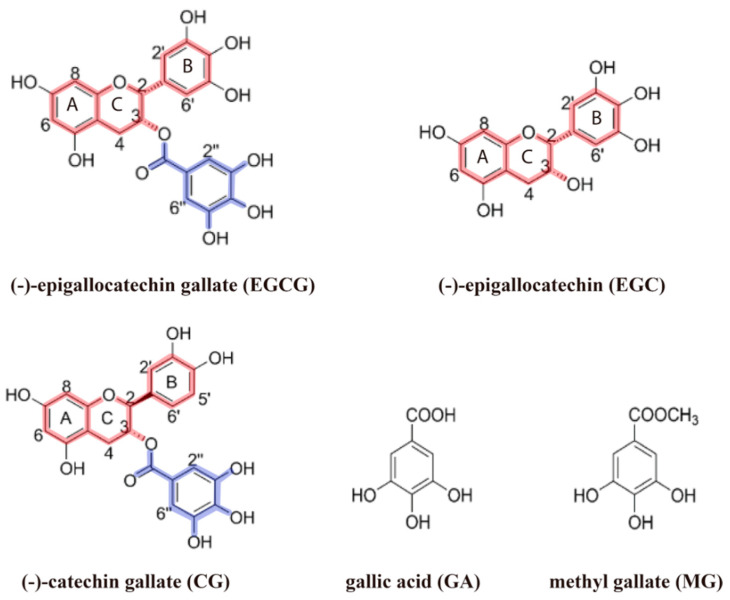
Catechin structural features critical for peptide oligomer binding. Highlighted in different colors are the structural determinants involved in the interaction of green tea catechins with Aβ_42_, PrP_106–126_, and AT3Q55 oligomers. The flavan-3-ol unit (red) is essential for the interaction with amyloid proteins, while the presence of the gallate moiety (blue) is not essential for binding, but it increases the affinity.

The investigation by Sironi et al. revealed not only new EGCG binding partners, but also the EGCG epitopes for binding protein oligomers [12]. The STD NMR data of Sironi et al. suggests that EGCG binding to Aβ_42_ and AT3Q55 involves all structural motifs of EGCG, because saturation transfer was observed not only for rings A, B, and C, which define the catechin backbone, but also for the esterified gallate ring (Figure 4; highlights). However, the degree of saturation transfer was not uniform for all four rings, but decreased in the order A > gallate ring > B > C. EGCG binding to PrP_106–126_ showed the lowest STD effect with protons H2’-H6’ and H2”-H6” displaying the most significant STD signal intensity. Although these observations suggested that both the catechin backbone and esterified gallate ring are important for binding to amyloid oligomers, previous studies claimed that only gallate forms of catechins show anti-amyloidogenic activity and that gallic acid was sufficient for exerting inhibition [101,102].

The authors thus evaluated whether (i) catechins lacking the gallate ester e.g., EGC, and (ii) the gallate moiety alone e.g., gallic acid (GA) and methyl gallate (MG), are capable of binding Aβ_42,_ PrP_106–126_, and AT3Q55 oligomers. Catechin gallate (CG), a minor component of green tea, was also assessed because its anti-amyloidogenic activity was unknown. STD experiments revealed that whereas both EGC and CG can interact with the aggregated forms of the three amyloid proteins, no observable STD signal was evident for GA and MG. These results suggest that the gallate moiety alone is insufficient for binding to amyloid oligomers.

To assess the contribution of the gallate moiety for binding to amyloid oligomers, Sironi et al. conducted competitive STD experiments aimed at gauging the relative binding affinities of EGCG, EGC, and CG for Aβ_42_, PrP_106–126_, and AT3Q55 oligomers. Compared to the gallate catechins EGCG and CG, EGC binds significantly weaker to the Aβ and PrP oligomers. Sironi et al. therefore concluded that although the gallate moiety is not essential for binding, it does, however, increase the affinity considerably (Figure 3).

### 4.2. Morphology of Amyloid Aggregates in the Presence of Catechins

Sironi et al. further investigated how EGCG, EGC, and CG affected the aggregation state of Aβ_42,_ PrP_106–126_, and AT3Q55. Using transmission electron microscopy (TEM) the authors characterized the morphology of the aggregates in the presence and absence of the catechins. EGCG, CG, and EGC reduced the formation of protofibrils and enabled the formation of amorphous aggregates. Although the effect of EGCG on Aβ was already known, Sironi et al. found that a similar mechanism applies also for CG and EGC. In contrast, when Aβ was incubated with MG and GA, no appreciable difference was observed between the treated and untreated fibrils, as expected based on the negligible binding of MG and GA to Aβ aggregates in STD experiments. These results were further corroborated by CD measurements, which showed a decrease in β-sheet content of the amyloid oligomers in the presence of EGCG and CG and negligible changes in the presence of GA and MG.

In general, Sironi et al. found that the flavan-3-ol moiety, i.e., rings A–C, of catechins is essential for the interaction with amyloid proteins. Moreover, whereas the gallate moiety alone is insufficient to bind amyloid oligomers, it significantly enhances the binding affinity and in turn the remodeling capacity (Figure 4). These observations will assist the rational design of new compounds with anti-amyloidogenic effects or of tools for studying the amyloid aggregation process in greater detail. Both approaches are described in the following sections.

## 5. Synthetic Polyphenol-Based Amyloid Inhibitors

**Figure 5:**
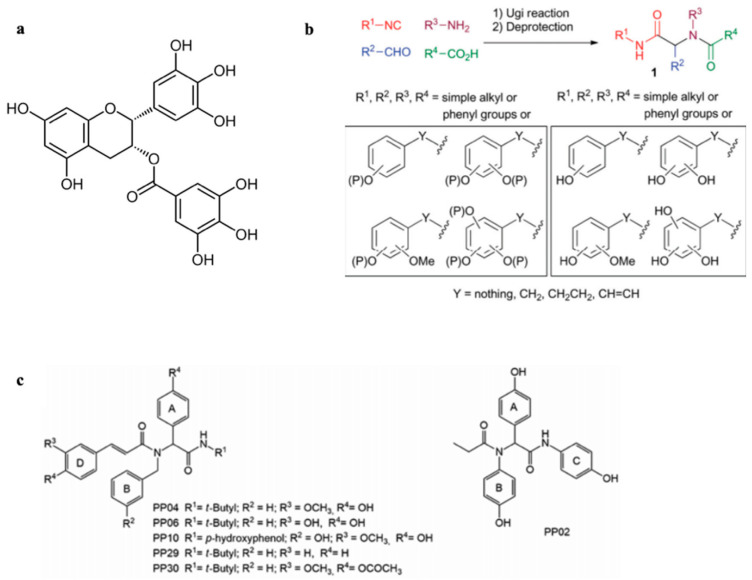
Synthetic mimics of natural polyphenols. Based on natural polyphenols such as EGCG (**a**), polyphenolic compounds were generated using a fragment-based synthesis (**b**) with monocyclic, phenol containing building blocks. (**c**) Synthetic compounds lacking the catechin phenolic rings [37] *Org. Biomol. Chem*. **2017**, *15*, 9331–9935. Published by The Royal Society of Chemistry [38] Reprinted (adapted) with permission from The American Chemical Society *Chem. Neurosci.* **2019**, *10*, 4462–4475. Copyright (2019) American Chemical Society.

Synthetic polyphenols are a potentially better therapeutic approach than their natural counterparts, as they may preserve a similar amyloid inhibitory potential but exhibit improved bioavailability. For example, Lambruschini et al. developed a library of complex synthetic polyphenols inspired by natural catechins (Figure 5b) [37]. Following this initial work, Tomaselli et al. investigated both in vitro and in vivo the most promising members of the library and their ability to interact and inhibit oligomeric Aβ [38]. They selected four polyphenolic compounds from the library based on their inhibitory efficacy and synthetized two additional new compounds lacking the catechin polyphenolic rings (Figure 5c). The six ligands were incubated with Aβ_42_ and fibrillization was evaluated using ThT. Notably, only the two new compounds lacking phenolic groups were unable to inhibit fibrillization. These results suggest that the phenolic rings play a key role in inhibiting Aβ fibrillation.

The authors further evaluated the neurotoxic effects of these phenol-based compounds ex vivo in primary hippocampal neurons. Two of the originally selected compounds were non-toxic to cells therefore their interaction with Aβ_42_ were further analyzed using NMR spectroscopy. ^1^H-^15^N HSQC NMR experiments revealed that the selected polyphenolic compounds bind preferentially oligomeric vs. monomeric Aβ_42_ and a combination of NMR and docking studies showed that this binding occurs mainly at the central hydrophobic core of Aβ. These results are in agreement with the previously discussed data reported for EGCG by Ahmed et al. [21].

To further evaluate the therapeutic potential of the polyphenolic ligands, the authors analyzed their neurotoxic effects in vivo using model mice. Only one of the polyphenols was able to counteract the detrimental effects of Aβ_42_ on memory and glial cell activation. It is notable that through the interrogation of a synthetic catechin-based small molecule library, Tomaselli et al. were able to find a new polyphenol mimic, which efficiently inhibits Aβ_42_ aggregation in vitro and is able to counteract oligomeric Aβ_42_ memory impairment in mice. Such compound may serve as a lead for new potential therapeutic agents in the treatment of AD.

## 6. Catechins as Tools to Probe the Structural Determinants of Amyloid Toxicity: Application to αSyn Oligomers

Catechins not only inform the development of new anti-amyloidogenic drugs, but they are also excellent tools to modulate and control the toxicity of amyloidogenic oligomers ex vivo [21,35,36,38]. In a recent study by Fusco et al., EGCG treatment was used to generate αSyn oligomers with low toxicity in cultured mammalian cells [35]. Structural comparison with untreated, toxic assemblies unveiled the molecular determinants underlying membrane binding and damage as well as cellular toxicity of αSyn oligomers.

### 6.1. Properties of Toxic vs. Non-Toxic αSyn Oligomers

Given that previous studies reported the capacity of EGCG to remodel toxic αSyn oligomers into non-toxic species [27,36], Fusco et al. prepared αSyn oligomers in the presence and absence of excess EGCG, which they denote as type A and type B oligomers, respectively [35]. Type A and B oligomers exhibit remarkably different capacities to disrupt lipid bilayers and induce cellular dysfunction. Although treatment of mammalian cells with type B oligomers results in significant enhancement in intracellular calcein release and decrease in mitochondrial activity, addition of type A oligomers produces a cellular phenotype similar to untreated cells. Notably, the cellular phenotypes induced by type B oligomers, but not type A, resembles to a large extent the pathophysiology observed in neuronal models of PD [103,104].

**Figure 6:**
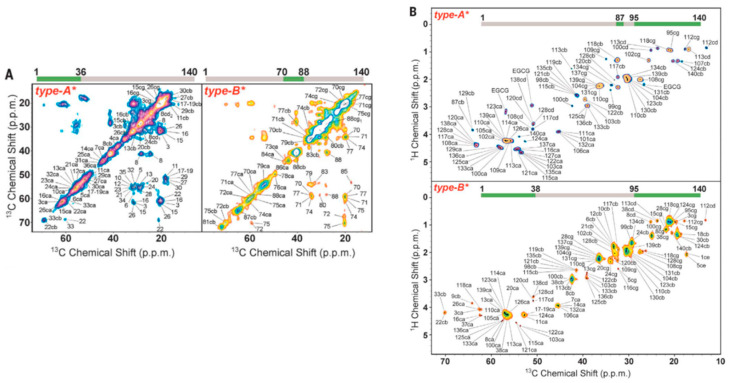
Solid-State NMR (ssNMR) spectra with magic angle spinning (MAS) of ⍺-syn oligomers. (**A**) ^13^C-^13^C dipolar-assisted rotational resonance (DARR) correlation spectra showing the aliphatic regions of type A (**left**) and type B (**right**) oligomers; (**B**) Insensitive nuclei enhanced by polarization transfer (INEPT) correlation spectra. The regions of the spectra that were detected are assigned with the respective residue number and highlighted in green in the bars on top. The labels ca, cb, cg, cd, and ce represent C^⍺^, C^β^, C^γ^, C^δ^, and C^ℇ^ atoms, respectively. Reprinted with permission from The American Association for the Advancement of Science. *Science* **2017**, *358*, 1440–1443. Copyright © 2017, Copyright © 2017 The Authors, some rights reserved; exclusive licensee American Association for the Advancement of Science.

To gain residue-resolution insight on the structural properties of αSyn oligomers that explain the type A vs. B differences in cellular toxicity, the authors turned to solid-state NMR (ssNMR) (Figure 6). ^13^C-^13^C dipolar-assisted rotational resonance (DARR) with magic angle spinning (MAS) was used to identify rigid regions in the αSyn oligomers. The DARR data revealed that type B oligomers contain a rigid β-sheet core spanning residues 70 to 88, whereas type A oligomers exhibit a rigid N-terminal segment spanning residues 3 to 36, yet with negligible secondary structure (Figure 6A). These observations were further complemented by Chemical Exchange Saturation Transfer (CEST) experiments, which showed substantial CEST broadening for the first 40 N-terminal residues in type A oligomers, indicating strong association of this region with the oligomeric core. For type B oligomers, significant CEST broadening was only observed for residues 70 to 79, i.e., a segment similar to the rigid region mapped through DARR.

**Figure 7:**
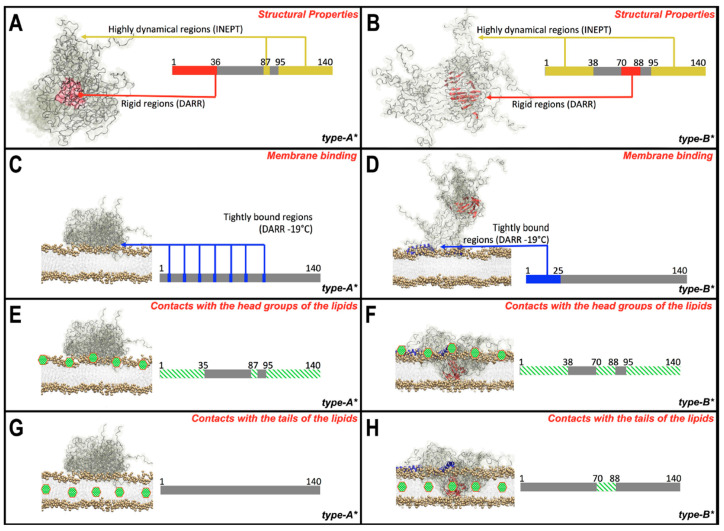
Properties of non-toxic and toxic ⍺Syn oligomers and their interactions with cell membranes. (**A**,**B**) Map of rigid vs. flexible regions in non-toxic type A (**A**) and toxic type B (**B**) oligomers. In the case of type B oligomers, the rigid region adopts a β-sheet secondary structure. (**C**,**D**) Interaction of type A (**C**) and type B (**D**) oligomers with the membrane, where the blue color represents regions within the oligomers that bind tightly to the lipid bilayer. In the case of type B oligomers (**D**) the regions tightly bound to the membrane contain an ⍺-helical secondary structure. (**E**,**F**) Interaction of type A (**E**) and type B (**F**) oligomers with the polar head groups of the membrane surface. (**G**,**H**) Type A oligomers (**G**) do not insert into the membrane, while type B (**H**) are able to insert into the bilayer through its rigid β-sheet rich core (red colored). Reprinted with permission from The American Association for the Advancement of Science. *Science* **2017**, *358*, 1440–1443. Copyright © 2017, Copyright © 2017 The Authors, some rights reserved; exclusive licensee American Association for the Advancement of Science.

To complement the identification of rigid regions in αSyn oligomers through ^13^C-^13^C DARR, the authors acquired insensitive nuclei enhanced by polarization transfer (INEPT) ssNMR data, which are sensitive to highly dynamic regions of proteins (Figure 6B). Both oligomers exhibited mobile C-terminal segments, but only in the case of type B oligomers was the N-terminus also flexible. The INEPT measurements were further corroborated by dot blot assays with primary antibodies recognizing distinct N- and C-terminal αSyn regions. Although both type A and type B oligomers exhibited significant cross-reactivity to C-terminal antibodies, type B oligomers showed a substantially larger reactivity to N-terminal antibodies compared to type A. Overall, these results suggest that toxic (Type B) αSyn oligomers contain a rigid β-sheet rich core and highly flexible N- and C-terminal segments (Figure 7A,B). These results suggest that loss of the β-sheet core element and rigidification of the N-terminus, as promoted through EGCG remodeling, reduces oligomer toxicity.

### 6.2. Interaction of Toxic and Non-Toxic αSyn Oligomers with Membranes

Given the strong association between membrane disruption and cellular toxicity observed for type A vs. type B oligomers, Fusco et al. elucidated at progressive degrees of resolution the interaction of both oligomer variants with biomimetic Small Unilamelar Vesicles (SUVs), to gain mechanistic insight on the structural basis of oligomer toxicity. Fluorescence correlation spectroscopy revealed that both types of oligomers bind tightly to the SUVs, but type B oligomers display a higher binding affinity. Moreover, confocal scanning microscopy images of primary cortical neurons further revealed that type B oligomers colocalize with the plasma membrane.

Solid-State Paramagnetic Relaxation Enhancement (PRE) NMR experiments with spin labels at the phospholipid headgroup or fatty acid tail were used to assess the degree of αS oligomer insertion. Both oligomer variants exhibited significant PRE-induced line broadening when the spin label was attached to the phospholipid headgroup, consistent with interactions with the membrane surface (Figure 7E,F). However, only type B oligomers were able to insert into the hydrophobic interior of the lipid bilayer, as indicated by the loss of intensity exclusively for type B oligomers when the spin label was attached to the fatty acid tail (Figure 7G,H). Notably, PRE experiments with ^1^H-^13^C INEPT readout revealed that the mobile regions of type B oligomers exhibit negligible peak broadening when the spin label is placed in the membrane interior, suggesting that the non-amyloid-β component (NAC) β-sheet core is the primary element embedded into the membrane (Figure 7H).

To further characterize the regions in the oligomers that bind tightly with the membrane the authors recorded ^13^C-^13^C DARR spectra in the presence of membranes. For type A oligomers it was found that the region tightly bound to the membrane lacks a defined structure (Figure 7C). On the other hand, for the type B variant selected residues from the N-terminal region were found to adopt an ⍺-helical conformation. These results indicate that the N-terminal region of toxic oligomers is responsible for promoting interactions with the membrane surface (Figure 7D). In support of these conclusions, substitution of alanine at position 30 for proline (αSyn_A30P_) and deletion of the N-terminal segment spanning residues 2 to 9 (αSyn_Δ2–9_) compromises membrane binding and plasma membrane colocalization and reduces cell toxicity relative to wildtype. Although the membrane detuning effect of A30P and Δ2–9 at the level of αSyn monomers were previously known [105,106,107], Fusco et al. showed that these observations also extend to αSyn oligomers.

Overall, this study exemplifies how catechins and more specifically, EGCG, can be used as a useful tool to generate structurally and functionally divergent oligomers and consequently to establish structure—toxicity relationships. Through the comparison of type A (EGCG-treated) vs. type B (untreated) αSyn oligomers, Fusco et al. elegantly revealed two essential structural elements critical for oligomer toxicity; namely an N-terminal lipophilic region that promotes membrane association and a β-sheet core which inserts into the membrane (Figure 7). In the following section, we provide another illustrative example of how catechins can be used to determine the structure—toxicity relationships for the AD associated Aβ oligomers.

## 7. Catechins as Tools to Probe the Structural Determinants of Amyloid Toxicity: Application to Amyloid Beta Oligomers

An emerging concept that reconciles the large body of literature reporting differing mechanisms of amyloid oligomer toxicity, is that toxicity cannot be attributed to any given Aβ species, but is a generic property arising from the exposure of “toxic surfaces” shared by multiple soluble Aβ assemblies (Aβ_n_) produced by the nucleation-dependent aggregation process [108,109]. However, these exposed toxic interfaces had remained largely unknown.

Ahmed et al. in 2019 provided a glimpse of such otherwise elusive toxic surfaces by using a catechin library to generate different Aβ oligomers with different degrees of toxicity [34]. A similar approach had been previously shown to be effective in revealing unique molecular features for other amyloidogenic systems such as HypF-N [110] and α-syn [35], described above. However, whereas previous studies used binary comparisons to discern structural differences contributing to oligomer toxicity, Ahmed et al. highlights the strength of using a library of oligomeric species to partition structural features using clustering analyses. By combining cell toxicity assays, electron microscopy, NMR spectroscopy, dynamic light scattering (DLS), wide-angle X-ray diffraction (WAXD), and fluorescence assays, Ahmed et al. identified a cluster of key toxicity determinants and their underlying mechanism of action.

### 7.1. Building an Aβ40 Oligomer Library Using Catechins

**Figure 8:**
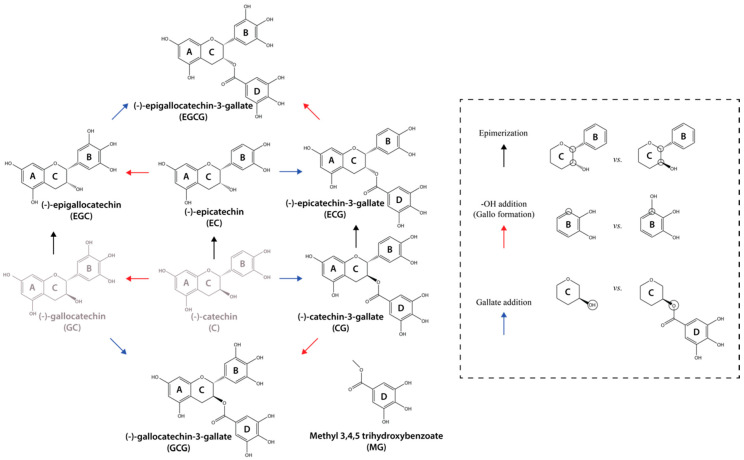
Catechin library used to modulate Aβ toxicity. Arrows represent the covalent modifications used to generate the catechin library and do not reflect endogenous synthetic pathways. The substitutions are identified as epimerization (black), 3′-OH addition to ring B (gallo formation) (red), and esterification of the ring C-OH by gallate addition (blue). The (−)-catechin and (−)-gallocatechin compounds (gray) were not included in the library and are shown here for comparative purposes [34]. *Chem. Sci.* **2019**, *10*, 6072–6082. Published by The Royal Society of Chemistry.

**Figure 9:**
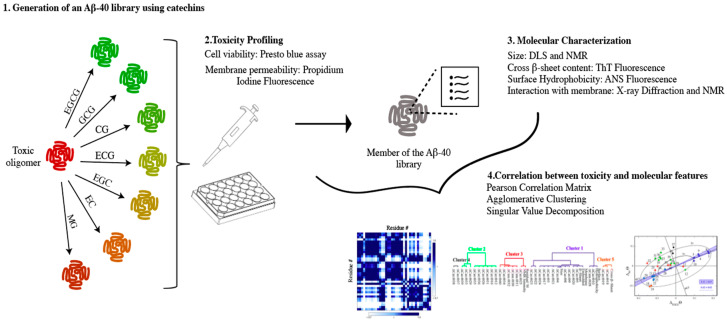
Experimental design to identify the toxicity determinants of Aβ_40_ oligomers. (**1**) A library of Aβ_40_ oligomers was prepared by incubating Aβ_40_ oligomers with a collection of seven catechins expected to remodel to varying extents their pre-existing toxic conformation into a less toxic conformation. (**2**) The toxicity of the different oligomers was evaluated by using Presto Blue assay, which relies on mitochondrial activity to inform on cell viability, and PI fluorescence, which reports if the cell membrane is being compromised. (**3**) Different techniques with different degrees of resolution were implemented to extensively characterize the molecular features of the oligomers present in the Aβ-library. (**4**) Correlation studies between toxicity (**2**) and molecular features (**3)** were necessary to reveal the key determinants of toxicity. Figure in (**4**) can be found in reference [34] *Chem. Sci.* **2019**, *10*, 6072–6082. Published by The Royal Society of Chemistry.

The catechin library used by Ahmed et al. to generate an ensemble of Aβ oligomers with different degrees of cytotoxicity includes EGCG and six EGCG analogs, with EGCG exhibiting the most potent detoxifying effect. For the generation of EGCG analogs three key modifications were used: epimerization, 3′-OH addition (gallo formation), and gallate addition (Figure 8). These three modifications were previously shown to influence the capacity of the catechins to interact with Aβ assemblies and the morphology of the resulting Aβ species [20], and therefore expected to generate Aβ assemblies with different cytotoxicities. After exposing Aβ oligomers to seven different catechins, the authors profiled their relative cytotoxicity using the PrestoBlue assay, which measures cell viability. This assay revealed that Aβ oligomers formed in the absence of catechins significantly reduce cell viability. However, when the Aβ assemblies were treated with catechins, the cells were more viable with a decreasing cytotoxicity gradient from epicatechin EC to EGC to EGCG. A similar ranking was observed when evaluating the potency of the catechins to recover the loss of cell membrane integrity induced by Aβ oligomers, as probed through propidium iodide fluorescence microscopy. Overall, these results clearly indicate that the soluble oligomers generated through the catechin library induce different levels of cytotoxicity and therefore their comparative analysis is likely to unveil the molecular features underlying the toxicity of Aβ soluble assemblies (Figure 9).

### 7.2. Molecular Characterization of the Aβ Oligomer Library

Given that for model amyloidogenic proteins one of the key toxicity determinants is oligomer size [93], Ahmed et al. measured the size distribution of the different Aβ oligomers present in their library. The authors found through ^1^H-NMR intensity-based experiments that the populations of monomeric Aβ species are reduced upon catechin addition. Furthermore, DLS experiments revealed that high molecular weight (HMW) NMR invisible Aβ assemblies exhibited a shift towards intermediate molecular weight aggregates in the presence of catechins. The extent of this size remodeling is catechin-dependent with some catechins such as CG and EGCG causing a large decrease in the populations of monomers and high molecular weight species, while others such as MG exhibit negligible changes.

The authors also evaluated whether the Aβ oligomer library exhibited variations in surface hydrophobicity and cross-β-sheet content, two key structural elements native to amyloid assemblies. Using 8-anilino-1-naphthalenesulfonic acid (ANS) and Thioflavin T (ThT) extrinsic fluorescent molecular probes as reporters of surface hydrophobicity and cross-β-sheet content, respectively, Ahmed et al. found that indeed both structural features are decreased upon catechin treatment, in a markedly catechin-dependent manner. Overall, these findings suggest that the catechin-modulated Aβ oligomer library exhibits variations in morphology, surface properties, and supramolecular structure.

### 7.3. Interaction of the Aβ Oligomer Library with Cell Membranes

**Figure 10:**
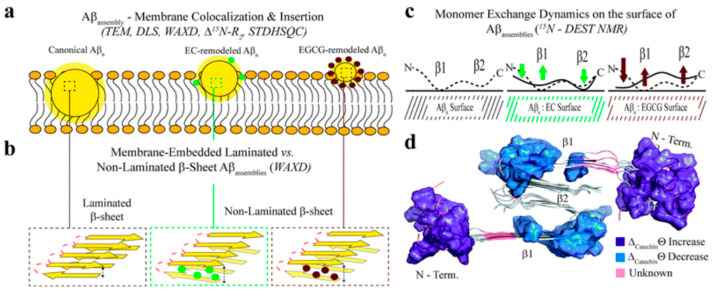
Proposed model for the molecular determinants of Aβ assembly toxicity. (**a**): Catechin-free oligomers (canonical Aβ_n_) insert and colocalize efficiently into the membrane due to their significant solvent exposure of hydrophobic surfaces. The catechin-remodeled oligomers with less exposed hydrophobic sites only insert into the membrane partially. (**b**): Both laminated and non-laminated cross-β-sheet structures can insert into the membrane, which indicates that cross-β-sheet structures are not required for membrane insertion. (**c**): The interaction of monomeric Aβ with toxic and remodeled oligomers is different within a membrane environment. EGCG-remodeled oligomers (maroon) show a significant disengagement with the β1 region and an opposite enhancement in the contacts with the N-terminal region compared to untreated (black) oligomers. The EC-remodeled (green) oligomers exhibit a pattern at the N-terminus and β1 regions intermediate to the canonical and EGCG-remodeled oligomers, while a further enhancement in C-terminal contacts relative to both canonical and EGCG-remodeled *Aβ_n_* is observed. (**d**): In the Aβ_40_ fibril structure (PDB code: 2LMN) the residues that correlate with toxicity (blue) in the N-terminal and β_1_ regions can be found in the exterior of the fibril structure, while the β_2_ region not linked with toxicity is inaccessible to the environment [34]. *Chem. Sci.* **2019**, *10*, 6072–6082. Published by The Royal Society of Chemistry.

Hydrophobic exposure is a well-known driver of membrane binding. In agreement with this notion, Ahmed et al., using a combination of electron microscopy, dynamic light scattering, and relaxation NMR measurements (i.e., ^15^N-DEST and R_2_), found that Aβ assemblies with higher degrees of surface hydrophobicity and toxicity interacted more tightly with the membrane compared to EGCG-treated oligomers (Figure 10a). Transmission electron microscopy (TEM) was first used to analyze the morphology of catechin-remodeled oligomers and their colocalization with SUVs. TEM revealed that the catechin-untreated oligomers colocalize with the membrane surface more effectively than the catechin-treated oligomers. A higher-resolution inspection of the interaction between Aβ oligomers and SUVs through solution NMR (i.e., ^15^N-transverse relaxation R_2_, ^1^H-STD, and ^15^N-DEST experiments) revealed specific Aβ regions critical for SUV binding. Residues 15–22 in the β_1_ region and 30–40 in the β_2_ region were shown to be crucial for Aβ-SUV interactions, while the N-terminal region (residues 1–12) appeared less engaged in such interactions. Moreover, WAXD experiments revealed how Aβ oligomers insert into the membrane and which conformation they adopt while inserted. In general, Ahmed et al. found that untreated Aβ oligomers in the membrane adopt a laminated cross β-sheet conformation. On the other hand, for catechin-treated oligomers the degree of insertion into the membrane is reduced and the oligomer β-sheet content ranks similarly to the cell toxicity assays, i.e., untreated oligomers > EC > EGCG (Figure 10a,b).

In the membrane environment toxic oligomeric Aβ recognizes Aβ monomers differently than remodeled assemblies. Toxic oligomers interact with Aβ monomers in the β_1_ and β_2_ regions; however, upon EGCG remodeling these contacts with the two β-strand sites are less engaged and instead there is a binding enhancement with the N-terminal region. EC-remodeled oligomers also showed, but to a lesser extent, an enhancement in the interaction with the N-terminal region and a disengagement with the β_1_ region. However, this is not observed for the β_2_ region (Figure 10c)

### 7.4. Correlation between Toxicity and Molecular Features of Aβ Oligomers

To identify which of the measured structural features correlates with toxicity, the simplest possible approach involves a series of direct linear correlations between each molecular feature, such as surface hydrophobicity, and toxicity. Although this approach might offer some initial insight, it does not consider molecular features that correlate with toxicity only collectively. A better method for selecting the Aβ assembly structural features relevant for toxicity is based on clustering analyses.

Ahmed et al. first used a correlation data matrix to identify groups of coupled molecular features. Using agglomerative clustering of the correlation data matrix, they were able to generate a dendrogram and divide the molecular observables into different clusters. They found a cluster of molecular features that correlates with toxicity. Those features pertain to oligomer size, surface hydrophobicity, membrane-embedded β-sheets, shielding of the N-terminus and simultaneous exposure of the β1 region to Aβ monomers (Figure 10).

Notably, the authors were able to validate the predictive power of the Aβ toxicity model by measuring the toxicity of other Aβ assemblies not used to train the model and comparing their results with those predicted by the model. A strong correlation (r ≥ 0.94) between the predicted and observed toxicities demonstrates the prognostic capacity of the model. However, it is important to clarify that correlation does not necessarily mean causation, and in some instances, a lack of correlation cannot be attributed to a lack of functional relevance. Nevertheless, the predictive properties of the model enabled by the catechin library may prove useful in evaluating the toxicities of other oligomers in the future.

## 8. Future Directions and Conclusive Remarks

The use of a targeted catechin library to elucidate the molecular determinants underlying the toxicity of soluble Aβ assemblies has opened new opportunities for the study of other amyloidogenic proteins. Since EGCG has been suggested to inhibit amyloid aggregation of most IDPs with a similar mechanism [39], it is possible that the molecular attributes correlating with toxicity in Aβ may be transferrable to other systems such as aSyn and IAPP. Similar catechin libraries could be used to test this hypothesis in other amyloidogenic systems different from Aβ by generating soluble oligomers with different degrees of toxicity. A clear understanding of what drives toxicity of soluble amyloid assemblies is necessary to develop therapeutic solutions for the prevention and treatment of neurodegenerative diseases and possibly other amyloidoses.

Although the therapeutic potential of EGCG, the catechin with the most potent oligomer detoxifying effect, is limited by its poor pharmacokinetics, understanding its interaction with toxic soluble assemblies formed by Aβ and other IDPs is anticipated to facilitate the design of more promising EGCG analogs. Such analogs may exhibit enhanced bioavailability and anti-amyloidogenic properties compared to EGCG. Alternatively, bioavailability can also be improved by combining catechins with dilute saponins [84]. The non-conventional use of catechins in the study of amyloidogenic diseases reviewed here illustrates how catechins serve as a valuable steppingstone towards the development of structure-toxicity correlations that will inform the design of future amyloid modulators.
